# miRNA Expression Signatures of Therapy Response in Squamous Cell Carcinomas

**DOI:** 10.3390/cancers13010063

**Published:** 2020-12-28

**Authors:** János Tibor Fekete, Ágnes Welker, Balázs Győrffy

**Affiliations:** 1 Department of Bioinformatics and 2nd Department of Pediatrics, Semmelweis University, H-1094 Budapest, Hungary; fekete.janos@med.semmelweis-univ.hu; 2 Research Center for Natural Sciences, Momentum Cancer Biomarker Research Group, Institute of Enzymology, Magyar tudósok körútja 2., H-1117 Budapest, Hungary; welker.agnes@hallgato.ppke.hu; 3 Faculty of Information Technology and Bionics, Pázmány Péter Catholic University, H-1083 Budapest, Hungary

**Keywords:** chemotherapy, cisplatin, carboplatin, cancer, miRNA, head and neck cancer, cervical cancer, lung cancer, logistic regression, receiver operator characteristics

## Abstract

**Simple Summary:**

miRNAs play role in various diseases and can also modulate therapy response. Our aim was to identify predictive miRNAs in platinum treated squamous cell carcinomas (SCC). Using a set of 266 squamous cancer samples we uncovered 16, 103, and 9 miRNAs correlated to chemotherapy response in the cervical, head and neck, and lung squamous cell carcinomas, respectively. By employing a logistic regression model, a signature comprising a set of six miRNAs was established capable to predict chemotherapy response with an AUC of 0.897. Our results show common molecular features of SCC tumors and pinpoint the most important miRNAs related to treatment outcome.

**Abstract:**

Introduction: Squamous cell carcinomas (SCC) are a major subgroup of malignant tumors with a platinum-based first-line systematic chemotherapy. miRNAs play a role in various diseases and modulate therapy response as well. The aim of this study was to identify predictive miRNAs in platinum-treated SCCs. Methods: miRNA expression data of platinum-treated head and neck (HNSC), cervical (CESC) and lung (LUSC) cancer were collected from the TCGA repositories. Treatment response was defined based on presence or absence of disease progression at 18 months. Responder and nonresponder cohorts were compared using Mann–Whitney and Receiver Operating Characteristic tests. Logistic regression was developed to establish a predictive miRNA signature. Significance was set at FDR < 5%. Results: The integrated database includes 266 SCC patient samples with platinum-based therapy and available follow-up. We uncovered 16, 103, and 9 miRNAs correlated to chemotherapy response in the CESC, HNSC, and LUSC cohorts, respectively. Eight miRNAs overlapped between the CESC and HNSC subgroups, and three miRNAs overlapped between the LUSC and HNSC subgroups. We established a logistic regression model in HNSC and CESC which included six miRNAs: hsa-miR-5586 (Exp (B): 2.94, *p* = 0.001), hsa-miR-632 (Exp (B): 10.75, *p* = 0.002), hsa-miR-2355 (Exp (B): 0.48, *p* = 0.004), hsa-miR-642a (Exp (B): 2.22, *p* = 0.01), hsa-miR-101-2 (Exp (B): 0.39, *p* = 0.013) and hsa-miR-6728 (Exp (B): 0.21, *p* = 0.016). The model using these miRNAs was able to predict chemotherapy resistance with an AUC of 0.897. Conclusions: We performed an analysis of RNA-seq data of squamous cell carcinomas samples and identified significant miRNAs correlated to the response against platinum-based therapy in cervical, head and neck, and lung tumors.

## 1. Introduction

The vast majority of malignant tumors have epithelial origin. These tumors are termed “carcinomas” and can be further divided into adenocarcinoma and squamous cell carcinoma. The most affected anatomical sites where squamous cell carcinoma (SCC) can occur include the skin, lung, the head and neck region, esophagus, cervix and thyroid gland. SCCs share common characteristics not only in their development but also in the course of the disease [1]. Similarities were also documented in gene expression, miRNA expression, and mutation profiles [2].

Worldwide, lung cancer is the most frequent tumor type with more than 2 million new cases [3], with particularly high incidence and mortality in some Central European countries [4]. Approximately 30% of lung cancer cases originate in squamous cells (LUSC) [5]. In the head and neck region tumors can develop in the lips, oral cavity, oropharynx, sinonasal cavities, larynx, hypopharynx, and salivary glands. Among these, head and neck squamous cell carcinoma (HNSC) is the most common type [6]. Cervical cancer is the fourth most common cancer in women, with higher incidence in low- and middle-income countries [7]. A common feature of these SCC tumors is the utilization of platinum compounds in their systemic chemotherapy. According to the protocols of the National Comprehensive Cancer Network [8], this can be based on either cisplatin or carboplatin.

The response rate of platinum based treatment varies by tumor type, and the mean proportion of responder is between 20 and 40% in non-small cell lung cancer [9], cervical cancer [10] and in head and neck cancer [11]. Another problem is that secondary resistance can develop in patients who were responders for the first line platinum treatment by the selection of resistant clones during treatment [12].

MicroRNAs (miRNAs) are small noncoding RNAs with a length of 22–25 nucleotides having a crucial role in posttranscriptional gene regulation. Changes in miRNA expression were linked to the pathogenesis of a wide range of diseases [13]. In cancer, affected miRNAs can regulate for example key cancer hallmark features including signaling pathways of cell proliferation, cell cycle and apoptosis. In addition, miRNAs can also suppress the expression of wild-type tumor suppressor genes [14]. Altered miRNA expression can also modulate therapy response. For example, miR-128 and miR-155 have been reported as key miRNAs related to platinum response in patients with non-small cell lung cancer [15], miR-200b and miR-155 were predictive biomarkers for the efficacy of chemoradiation in head and neck cancer patients [16] and a total of 25 differentially expressed miRNAs have been linked to response to acetoxychavicol acetate (ACA) and/or cisplatin in human cervical carcinoma cells [17].

The aim of this study was to identify miRNAs which could serve as predictive biomarkers in platinum-treated SCCs. Our secondary goal was to detect overlapping miRNA expression patterns in platinum-treated head and neck, cervical and lung squamous cell carcinoma samples.

## 2. Results

Overall, 1309 (CESC: 307, HNSC: 524, LUSC: 478) patients with squamous cell carcinomas were identified in the GDC data portal. From these patients, we excluded those with insufficient clinical data or without a platinum-based (cisplatin or carboplatin) chemotherapy. Finally, altogether 266 patients were retained for the analysis: 94 patients in the CESC subgroup (16 nonresponder and 78 responder), 105 patients in the HNSC subgroup (34 nonresponder and 71 responder) and 67 patients in the LUSC subgroup (16 nonresponder and 51 responder). Aggregate clinical characteristics of the samples are presented in Table 1.

The expression dataset contains information for 1881 miRNAs. From these, we had retained 599 miRNAs with non-zero expression in the more than 50% of the analyzed samples.

### 2.1. miRNAs Associated with Treatment Outcome in Each SCC Cohort

**Figure 1:**
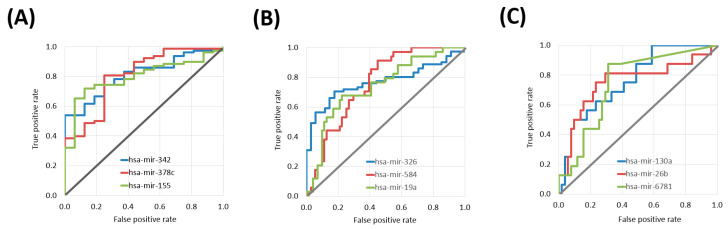
ROC curves of the top three miRNAs with predictive power in platinum treatment in different tumor tissues including cervical cancer samples (**A**), head and neck cancer (**B**), and lung squamous cell carcinoma (**C**).

Sixteen differentially expressed miRNAs were identified in the CESC cohort. The top three miRNAs were hsa-miR-342 (Mann–Whitney test *p* value: 9.68 × 10^−5^, AUC: 0.811), hsa-miR-378c (*p* value: 1.29 × 10^−4^, AUC: 0.805) and hsa-miR-155 (*p* value: 2.01 × 10^−4^, AUC: 0.796) (Table 2 and Figure 1A).

In the HNSC cohort 103 differentially expressed miRNAs associated with treatment outcome. The top three miRNAs were hsa-miR-326 (*p* value: 9.24 × 10^−6^, AUC: 0.768), hsa-miR-584 (*p* value: 2.02 × 10^−5^, AUC: 0.758) and hsa-miR-19a (*p* value: 4.66 × 10^−5^, AUC: 0.742) (Table 3 and Figure 1B).

There were nine differentially expressed miRNAs in the LUSC samples. The top three miRNAs were hsa-miR-130a (*p* value: 1.61 × 10^−3^, AUC: 0.763), hsa-miR-26b (*p* value: 2.64 × 10^−3^, AUC: 0.751) and hsa-miR-6781 (*p* value: 1.87 × 10^−3^, AUC: 0.744) (Table 4 and Figure 1C).

When investigating all three tumor types, there were in total 128 miRNAs with statistically significant differences in expression between nonresponder and responder cohorts in at least one subgroup. Out of these significant miRNAs, 11 miRNAs had significant differences in at least two subgroups: three in the LUSC and HNSC subgroups (hsa-miR-130a, hsa-miR-15a, and hsa-miR-877) and eight in the CESC and HNSC subgroups (hsa-miR-142, hsa-miR-150, hsa-miR-16-1, hsa-miR-181b-1, hsa-miR-378a, hsa-miR-378c, hsa-miR-378d-2, and hsa-miR-5586). We could not find a miRNA significant in both the LUSC and CESC subgroups.

### 2.2. miRNA Expression Comparison between Tumor Types

**Figure 2:**
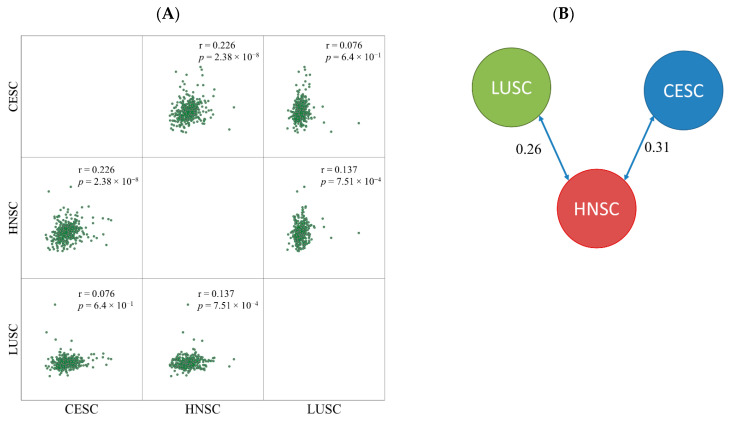
Pairwise comparison of fold changes by Pearson correlation (**A**) and similarity index determined by clustering (**B**).

Based on the log2 expression fold change between nonresponder and responder cohorts the samples were divided into two clusters. CESC and HNSC formed one cluster and LUSC samples were separated into a different node. The similarity score was 0.31 between CESC and HNSC and 0.26 between LUSC and HNSC samples (Figure 2). The correlation between HNSC and LUSC was much lower (0.13).

### 2.3. Logistic Regression Based Classification Model

We combined the HNSC and CESC samples to have a sufficient sample number for the development of a classification model capable of predicting chemotherapy response. The selection of HNCS and CESC was based on their higher similarity scores (due to the differences between HNSC and LUSC the overall model did not improve when including LUSC samples as well). The samples were randomly divided into a training (70%) set and a test set (30%) multiple times. Thirty differentially expressed miRNA were identified in the HNSC and CESC merged samples, with an AUC over 0.66 and an FDR below 0.05.

In the second step a stepwise logistic regression with forward selection was performed to estimate the predictive power. After this second feature selection we retained nine miRNAs with the strongest link to treatment response. The accuracy of the model was 0.88 (95% CI: 0.81–0.93), the sensitivity was 0.69, and the specificity was 0.94 in the training set. In the test set the accuracy was 0.81 (95% CI: 0.69–0.90), the sensitivity was 0.60, and the specificity was 0.89. Overall, the 10-fold cross-validated model accuracy was 0.85 (95% CI: 0.79–0.90), the sensitivity was 0.60, and the specificity was 0.94.

**Figure 3:**
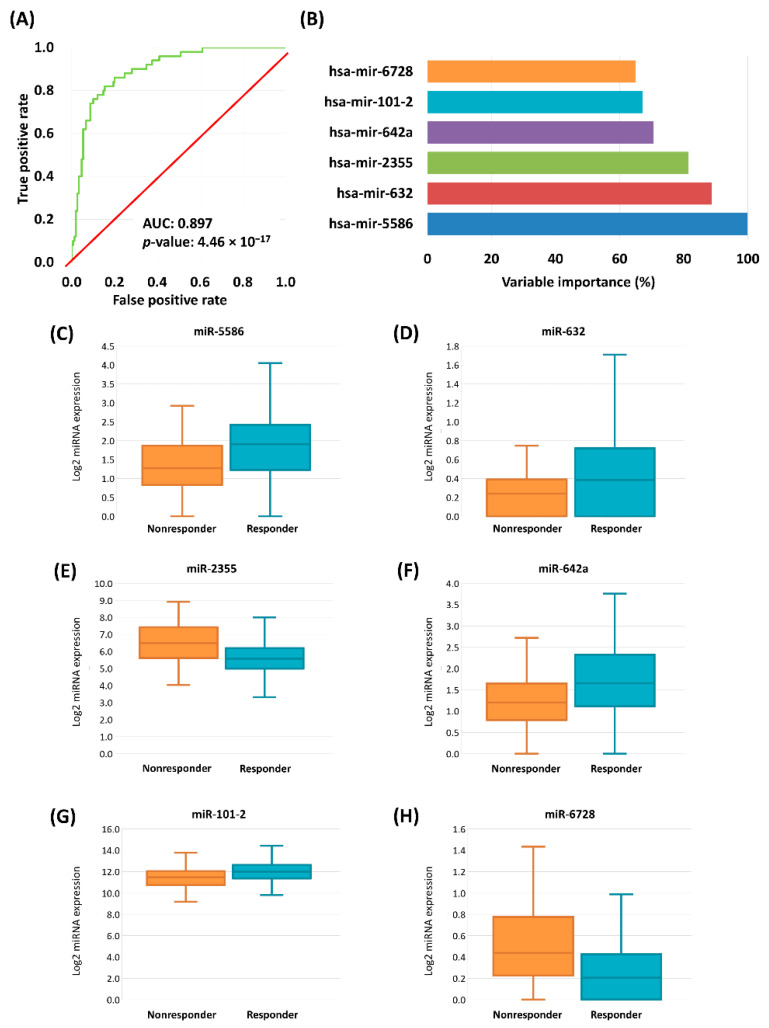
ROC curve of cross-validated logistic regression model of the chemotherapy-response signature in HNSC and CESC samples (**A**). Variable importance determined by logistic regression, miRNAs with significant result listed (**B**). Boxplots of the expression of significant genes in HNSC and CESC samples (**C**–**H**).

In the final step we simultaneously included all nine miRNAs with a significant impact. The significant remaining miRNAs were hsa-miR-5586 (Exp (B): 2.94, 95% CI for exp[B]: 1.65–5.51, *p* = 0.001), hsa-miR-632 (Exp (B): 10.75, 95% CI for exp[B]: 2.34–49.37, *p* = 0.002), hsa-miR-2355 (Exp (B): 0.48, 95% CI for exp[B]: 0.29–0.79, *p* = 0.004), hsa-miR-642a (Exp (B): 2.22, 95% CI for exp[B]: 1.21–4.07, *p* = 0.01), hsa-miR-101-2 (Exp (B): 0.39, 95% CI for exp[B]: 0.18–0.82, *p* = 0.013), and hsa-miR-6728 (Exp (B): 0.21, 95% CI for exp[B]: 0.06–0.75, *p* = 0.016) (Figure 3). In this final model hsa-miR-181b-2, hsa-miR-26-2, and hsa-miR-584 were not significant (Table 5).

### 2.4. Target Gene Prediction

Since some miRNAs were over-expressed and other under-expressed, the overall effect of the combination of these could neutralize each other. For this reason we investigated the over- and under-expressed miRNAs separately. The analysis of miRNAs overexpressed in nonresponder group revealed that hsa-mir-2355 and hsa-mir-6728 have two common predicted target genes which are repeated in different KEGG pathways, ENTPD5 and NT5C3A. Both genes take parts in purine (hsa00230, *p* = 6.47 × 10^−10^) and pyrimidine metabolism (hsa00240, *p* = 5.20 × 10^−8^) and NTC3A has a role in nicotinate and nicotinamide metabolism (hsa00760) and other metabolic pathways (hsa01100) as well. The underexpressed miRNAs (hsa-mir-5586, hsa-mir-642a, hsa-mir-101, hsa-mir-632) have nine common predicted target genes in four KEGG pathways. Most of the predicted genes are overlapped between pathways (Table 6)

## 3. Discussion

In our study our aim was to explore the miRNAome of platinum-treated head and neck, cervical and lung squamous cell carcinoma samples. First, we determined the most significant therapy-response related miRNAs in each SCC cohort. In CESC the most significant gene was hsa-miR-342. Hsa-miR-342 is known to be downregulated in cervical cancer tissues and cell lines and suppressed proliferation, growth, invasion and migration in human cervical cells [18]. In the HNSC samples hsa-miR-326 had the best discriminatory ability between responder and nonresponder cohorts. hsa-miR-326 was previously characterized as a tumor suppressor in breast [19] and colorectal cancer [20]. In our analysis responder samples had significantly elevated expression of hsa-miR-326 compared to nonresponder samples. Finally, in the LUSC subgroup hsa-miR-130a was the most significant miRNA. Elevated expression of hsa-miR-130a increased resistance to platinum chemotherapy [21]. Our results are in line with these previous findings, as we also observed higher expression in the nonresponding cohort.

We examined the overlapping genes, e.g., genes significant in multiple cohorts. In HNSC and CESC groups we uncovered 8 common miRNAs with significant discriminatory ability between responder and nonresponder cohorts, whereas we found only three common significant miRNAs in the LUSC and HNSC samples. A potential explanation for the higher number of overlapping miRNAs in the HNSC and CESC group can be that HPV infection has a role in the etiology of both tumor types. We have found 4 significant miRNAs (hsa-miR-16, hsa-miR-145, hsa-miR-199b) which have been previously reported as HPV core miRNAs in HNSC and CESC clinical samples [22].

The eight overlapping miRNAs between HNSC and CESC groups were previously discussed as genes related to cancer. High plasma level of hsa-mir-142 were reported as a HPV-independent prognostic marker of combined radio-chemotherapy in HNSC patients [23], and its expression correlated with the inhibition of cell proliferation and chemoresistance in ovarian cancer cell lines [24]. Induced overexpression of hsa-mir-150 inhibited cell invasion and metastasis in ovarian cancer [25]. Inhibited proliferation and enhanced therapeutic effect of cisplatin was reported in osteosarcoma for hsa-mir-16 [26]. hsa-mir-181 was overexpressed in cisplatin resistant NSCLC cells [27], and correlated with worse survival in oral squamous cell carcinoma patients [28]. Finally, higher expression of hsa-mir-378 reversed chemoresistance to cisplatin in NSCLC cells [29]. In our analysis, all of these miRNAs except for hsa-mir-181b-1 were overexpressed in responders.

The three significant miRNAs shared between LUSC and HNSC (hsa-miR-130a, hsa-miR-15a, hsa-miR-877) are all upregulated in nonresponder samples. hsa-miR-130a was upregulated in esophageal squamous carcinoma cell lines [30] and in clear cell carcinoma of ovary patients and the overexpression of the miRNA was a biomarker for disease recurrence [31]. hsa-miR-130a was also elevated in cisplatin resistant ovarian cell lines [32]. hsa-miR-15 was the first miRNA linked to tumor suppression in cancer [33]. As a contrary, it has been reported that overexpression of hsa-miR-15 predicts poor disease-free survival and overall survival in colorectal cancer patients [34], and it promotes neuroblastoma migration [35]. hsa-miR-877 was reported as oncogene in gastric cancer [36], but another study found that it has a tumor suppressor role in prostate cancer [37]. Our result suggests that the higher expression of hsa-miR-130a, hsa-miR-15a, and hsa-miR-877 are all biomarkers of resistance.

In the last step we determined a miRNA signature capable of predicting the outcome of platinum-based chemotherapy in HNSC and CESC samples. Our model reached a high accuracy in the 10-fold cross-validation of 0.854. The final model includes six miRNAs with the strongest effect—the most significant of these was hsa-miR-5586, which was studied in diffuse large B-cell lymphoma patients and the elevated expression was associated with better outcome [38]. In our study the expression of mir-5586 was elevated in responder samples both in HNSC and CESC subgroups. The second most important variable in our model was hsa-miR-632 and responders had higher expression. Earlier, its upregulation was associated with better survival in colorectal cancer [39] and its downregulation had an inhibitory effect on hepatocellular carcinoma cell proliferation and invasion [40]. The expression of hsa-miR-2355 was higher in nonresponder samples of HNSC and CESC subgroups. In esophageal squamous carcinoma cell line Zhang et al. reported that overexpressed hsa-miR-2355 promoted cell proliferation and invasion [41].

In the last step target gene prediction was performed with DIANA-Tool for six miRNAs significant in the logistic regression model. For the hsa-mir-2355 and the hsa-mir-6728 we determined ENTPD5 and NT5C3A genes as potential targets. The ENTPDT gene was examined in numerous studies and association between higher expression of ENTPDT and chemotherapy response were reported in colorectal cancer cells [42] and prostate cancer [43]. In lung cancer cells decreased apoptosis rate was observed after knockdown of ENTPDT [44]. Interestingly, the most significant pathway related to genes targeted by the four miRNAs with lower expression (hsa-mir-5586, hsa-mir-642a, hsa-mir-101, hsa-mir-632) was the circadian entrainment pathway with six potential target genes. This pathway was demonstrated to be associated with platinum treatment resistance in cancer [45].

There is an important limitation of our study: we had only a restricted number of samples available for this analysis. Not only is the total number of SCC samples published low, but an important further restriction was the administration of a platinum-based protocol. Although we were able to validate the result of our final model using separate test/training cohorts in a ten-fold cross-validation, for the generalization of our results it will be necessary to repeat the analysis in the future using more clinical samples.

## 4. Materials and Methods

### 4.1. Database Construction

**Figure 4:**
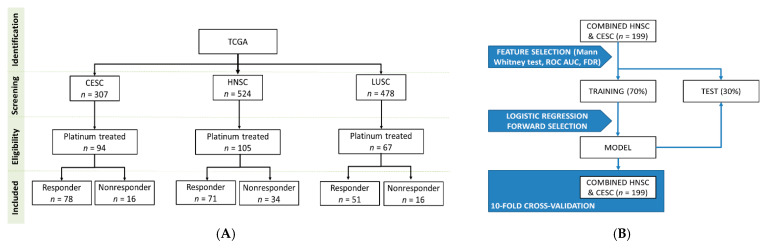
Overview of the database setup (**A**) and the analysis pipeline (**B**).

The level 3 miRNA expression data and the clinical information of samples were downloaded from GDC data portal [46]. All samples were primary tumors, cervical squamous cell carcinoma (CESC), head and neck squamous cell carcinoma (HNSC) or lung squamous cell carcinomas (LUSC). We retained only samples which were treated with cisplatin or carboplatin and had clinical information with survival times available (Figure 4A).

### 4.2. Determination of miRNAs with Best Discriminatory Ability by Tumor Types

For the analysis we utilized the log2 transformed reads per million mapped reads and filtered the datasets to include only miRNA expressed at a non-zero level in at least 50% of the samples. Based on the presence or absence of disease progression at 18 months after surgery we splitted patients into two cohorts. Patients who had any disease progression or death within 18 months were categorized as nonresponders and patients without a disease progression were considered as responders.

We compared nonresponder and responder cohorts across all genes using Mann–Whitney *U*-test and Receiver Operating Characteristic test in the R statistical environment [47] using Bioconductor libraries [48]. To avoid false discovery, we calculated false discovery rate (FDR) with the qvalue R package.

### 4.3. Similarity Detection between Tumor Types

To uncover similarities among tumor types, the log2 transformed fold change values of CESC, LUSC, and HNSC samples were converted into a ranked matrix. To obtain a ranked matrix and clustering we used the rankerGUI website [49]. The matrix was processed further to obtain a similarity score between tumor types and clustered them based on these similarity scores.

### 4.4. Model Building

As an initial feature selection step, we compared nonresponder and responder samples again with Mann–Whitney U-test and Receiver Operating Characteristics using a combined cohort comprising all HNSC and CESC samples. We retained only miRNAs if the *p*-value in the Mann Whitney test was below 0.01 and the FDR was below 5%, and the area under the curve in the Receiver Operating Characteristic (ROC) analysis was greater than 0.65. In the next step we performed a stepwise logistic regression model with forward selection as a second feature selection step to identify potential miRNA predictors of cisplatin/carboplatin sensitivity. The dependent variable was the responder status at 18 months, and the independent variables were the selected miRNAs from the previously selected miRNAs. In the model building process, the samples were randomly divided into a training (70%) and a test set (30%). Classification was based only on the training set. To avoid bias related to sample splitting we also performed a 10-fold cross-validation. To evaluate the overall performance of the logistic regression model we performed Receiver Operating Characteristic analysis to determine the area under curve (AUC). For the analysis the caret R package was used (Figure 4B).

### 4.5. KEGG Target Gene Prediction

For miRNAs significant in the logistic regression model we performed KEGG target prediction with the online DIANA-mirPath v3.0 by employing the microT-CDS algorithm [50]. For the pathway designation Fischer’s exact test was applied with a threshold of *p* ≤ 0.05 and a false discovery rate cutoff of 5% (FDR).

## 5. Conclusions

In summary, we performed an analysis of RNA-seq data of 266 squamous cell carcinomas samples and identified significant miRNAs correlated to the response against platinum-based therapy in cervical, head and neck, and lung tumors. We constructed a predictive model for HNSC and CESC with six significant miRNAs capable of predicting response to platinum-based treatment. Our results support the hypothesis of common molecular features of SCC tumors and pinpoint the most important miRNAs related to treatment outcome.

## Figures and Tables

**Table 1:** Overview of analysis squamous cancer cell datasets.

Characteristic	Cervical	Head and Neck	Lung	Total
Age (years ± standard deviation)	49.74 ± 12.50	57.12 ± 10.10	63.15 ± 9.56	56.03 ± 12.04
Gender (male/female)	0/94	90/15	50/17	140/126
Stage (unknown/I/II/III/IV)	94/-/-/-/-	27/1/3/8/66	1/11/35/18/2	122/12/38/26/68
Grade (unknown/I/II/III/IV)	8/3/46/36/1	6/9/57/26/7	67/-/-/-/-	81/12/103/62/8
Follow-up (months ± standard deviation)	47.64 ± 39.21	32.21 ± 21.39	35.87 ± 21.00	39.68 ± 30.44

**Table 2:** Top ten miRNA biomarker candidates of platinum-based therapy in cervical cancer (CESC).

CESC (*n* = 94)	Mean (Nonresponder)	Mean (Responder)	Fold Change	Mann–Whitney *p*	AUC	AUC (*p*-Value)	FDR
hsa-miR-342	6.390	7.445	1.17	9.68 × 10^−5^	0.811	8.30 × 10^−11^	1.1 × 10^−5^
hsa-miR-7702	0.397	1.413	3.56	1.11 × 10^−4^	0.808	1.90 × 10^−7^	1.8 × 10^−4^
hsa-miR-378c	2.226	3.293	1.48	1.29 × 10^−4^	0.805	3.60 × 10−^7^	3.5 × 10^−4^
hsa-miR-155	7.603	8.896	1.17	2.01 × 10^−4^	0.796	4.40 × 10^−9^	1.1 × 10^−5^
hsa-miR-378a	8.302	9.693	1.17	3.22 × 10^−4^	0.787	8.00 × 10^−6^	6.0 × 10^−3^
hsa-miR-502	3.118	3.624	1.16	1.61 × 10^−3^	0.752	9.10 × 10^−4^	1.2 × 10^−1^
hsa-miR-150	7.926	9.468	1.19	1.91 × 10^−3^	0.748	4.00 × 10^−5^	2.9 × 10^−2^
hsa-miR-5586	1.128	1.784	1.58	2.12 × 10^−3^	0.745	7.40 × 10^−6^	5.6 × 10^−3^
hsa-let-7g	8.820	9.226	1.05	2.85 × 10^−3^	0.738	2.30 × 10^−4^	7.5 × 10^−2^
hsa-miR-378d-2	0.299	0.600	2.01	5.72 × 10^−3^	0.72	1.40 × 10^−3^	1.4 × 10^−1^

**Table 3:** Top ten miRNA biomarker candidates of platinum-based therapy in head and neck cancer (HNSC).

HNSC (*n* = 105)	Mean (Nonresponder)	Mean (Responder)	Fold Change	Mann–Whitney *p*	AUC	AUC (*p*-Value)	FDR
hsa-miR-326	1.366	2.312	1.69	9.24 × 10^−6^	0.768	1.80 × 10^−9^	1.3 × 10^−5^
hsa-miR-584	7.750	6.430	1.21	2.02 × 10^−5^	0.758	1.20 × 10^−8^	1.5 × 10^−5^
hsa-miR-4791	0.275	0.809	2.94	4.66 × 10^−5^	0.742	2.80 × 10^−7^	1.7 × 10^−4^
hsa-miR-19a	5.587	4.705	1.19	8.11 × 10^−5^	0.739	2.50 × 10^−6^	6.4 × 10^−4^
hsa-miR-3610	0.659	1.100	1.67	8.54 × 10^−5^	0.738	3.60 × 10^−6^	8.0 × 10^−4^
hsa-miR-632	0.226	0.567	2.51	1.11 × 10^−4^	0.732	4.30 × 10^−7^	2.2 × 10^−4^
hsa-miR-5091	0.291	0.600	2.06	1.39 × 10^−4^	0.729	2.60 × 10^−6^	6.6 × 10^−4^
hsa-miR-3130-1	1.162	2.019	1.74	1.50 × 10^−4^	0.729	1.10 × 10^−5^	1.6 × 10^−3^
hsa-miR-4745	0.464	0.179	2.6	8.37 × 10^−5^	0.724	1.60 × 10^−5^	2.1 × 10^−3^
hsa-miR-143	14.970	15.801	1.06	2.59 × 10^−4^	0.721	1.40 × 10^−5^	1.9 × 10^−3^

**Table 4:** Top ten miRNA biomarker candidates of platinum-based therapy in lung squamous carcinoma (LUSC).

LUSC (*n* = 67)	Mean (Nonresponder)	Mean (Responder)	Fold Change	Mann–Whitney *p*	AUC	AUC (*p*-Value)	FDR
hsa-miR-130a	7.168	6.496	1.1	1.61 × 10^−3^	0.763	2.30 × 10^−5^	2.5 × 10^−2^
hsa-miR-26b	9.977	9.315	1.07	2.64 × 10^−3^	0.751	1.00 × 10^−3^	1.3 × 10^−1^
hsa-miR-6781	0.626	0.259	2.42	1.87 × 10^−3^	0.744	9.60 × 10^−5^	5.2 × 10^−2^
hsa-miR-361	8.940	8.398	1.06	4.64 × 10^−3^	0.737	1.60 × 10^−3^	1.6 × 10^−1^
hsa-miR-877	2.844	1.998	1.42	6.09 × 10^−3^	0.729	1.40 × 10^−3^	1.5 × 10^−1^
hsa-miR-30c-2	8.583	7.958	1.08	6.66 × 10^−3^	0.727	7.70 × 10^−4^	1.2 × 10^−1^
hsa-miR-335	7.650	6.918	1.11	7.94 × 10^−3^	0.722	2.40 × 10^−4^	7.6 × 10^−2^
hsa-miR-30c-1	8.484	7.834	1.08	8.66 × 10^−3^	0.719	1.30 × 10^−3^	1.4 × 10^−1^
hsa-miR-3193	0.207	0.541	2.62	9.20 × 10^−3^	0.713	1.90 × 10^−3^	1.7 × 10^−1^
hsa-miR-15a	7.618	7.172	1.06	1.12 × 10^−2^	0.712	3.10 × 10^−4^	8.4 × 10^−2^

**Table 5:** miRNAs significant in a logistic regression model combining CESC and HNSC samples.

MIR	B	SE	ExpB	95% CI for ExpB	*p* Value
hsa-miR-5586	1.077	0.321	2.937	1.565–5.511	0.001
hsa-miR-632	2.375	0.778	10.751	2.341–49.372	0.002
hsa-miR-2355	−0.732	0.255	0.481	0.292–0.793	0.004
hsa-miR-642a	0.798	0.309	2.222	1.213–4.069	0.010
hsa-miR-101-2	−0.946	0.38	0.388	0.184–0.817	0.013
hsa-miR-6728	−1.564	0.648	0.209	0.059–0.745	0.016
hsa-miR-181b-2	−0.438	0.267	0.645	0.383–1.089	0.101
hsa-miR-26a-2	0.922	0.479	2.513	0.982–6.429	0.054
hsa-miR-584	−0.118	0.163	0.888	0.646–1.222	0.467
Constant	8.578	4.694	5315.361	-	0.068

**Table 6:** The target genes of miRNAs with lower expression in the nonresponder cohort (hsa-mir-5586, hsa-mir-642a, hsa-mir-101, hsa-mir-632) predicted by DIANA-mirPath.

KEGG Pathway	*p*-Value	Number of Genes	Predicted Genes
Circadian entrainment (hsa04713)	2.17 × 10^−6^	6	FOS; GNB1; CREB1; GNAQ; GRIN2A; GRIN2B
Amphetamine addiction (hsa05031)	6.15 × 10^−5^	4	FOS; CREB1; GRIN2A; GRIN2B
Dopaminergic synapse (hsa04728)	0.009603	7	FOS; GSK3B; GNB1; CREB1; GNAQ; GRIN2A; GRIN2B
AMPK signaling pathway (hsa04152)	0.04017	3	CREB1; PPARGC1A; FOXO1

## Data Availability

The data used for the analyses were obtained from GDC data portal (https://portal.gdc.cancer.gov/) on 15/10/2020.
